# Effect of Bifunctional Montmorillonite on the Thermal and Tribological Properties of Polystyrene/Montmorillonite Nanocomposites

**DOI:** 10.3390/polym11050834

**Published:** 2019-05-08

**Authors:** Chengcheng Yu, Yangchuan Ke, Xu Hu, Yi Zhao, Qingchun Deng, Shichao Lu

**Affiliations:** Nanotechnology Center of Energy Resources, CNPC Nanochemistry Key Laboratory, College of Science, China University of Petroleum, Beijing 102249, China; yccycc9999@163.com (C.Y.); xuhu0001@126.com (X.H.); yccycc9999@126.com (Y.Z.); qingchundeng@163.com (Q.D.); shichaolu@yeah.net (S.L.)

**Keywords:** doubly functionalized montmorillonite, polystyrene, soap-free emulsion polymerization, thermal stability, tribological property

## Abstract

In this work, the effect of doubly functionalized montmorillonite (MMT) on the structure, morphology, thermal, and tribological characteristics of the resulting polystyrene (PS) nanocomposites was investigated. The modification of the MMT was performed using a cationic surfactant and an anionic surfactant or a silane coupling agent to increase the compatibility with PS matrix. The polystyrene/organo-montmorillonite (PS/OMMT) nanocomposite particles were prepared by soap-free emulsion polymerization. The OMMT was studied using Fourier-transform infrared (FTIR), X-ray diffraction (XRD), and thermogravimetric analysis (TGA). The structural and morphological changes of PS/OMMT nanocomposites were further characterized by dynamic light scattering (DLS), scanning electron microscopy (SEM), and transmission electron microscopy (TEM). The thermal stability of all the PS/OMMT nanocomposites was higher than that of the pure PS. The anti-wear properties of the polyalphaolefin (PAO) were significantly improved due to the introduction of the PS/OMMT nanocomposite particles. The nanocomposites prepared by a cationic surfactant and a silane coupling agent exhibited the best thermal stability and tribological performance. Our results provide the valuable insights needed to guide the design of lubrication and friction reducing materials.

## 1. Introduction

Clay-polymer nanocomposites have received great attention in recent years due to their exceptional properties, such as increased thermal stability, high fire resistance, and enhanced mechanical characteristics, compared with traditional polymer composites [1,2]. The improvement of many properties of clay-polymer nanocomposites could be achieved using a relatively low clay loading (usually < 10 wt %) in a polymer matrix [3]. Among the layered silicates, montmorillonite (MMT) was one of the most widely utilized to fabricate clay-polymer nanocomposite, mainly because the MMT was easily available with a high aspect ratio and high expansion capacity [4]. The unit crystal structure of the MMT consisted of two silicon tetrahedral sheets and a central sheet of alumina octahedrons. The negative charge was generated on the surfaces of the MMT layers due to the isomorphic substitutions of lower valance ions (Mg^2+^, Fe^2+^) for the central atoms (Al^3+^, Si^4+^) in the interior crystal layers of the MMT [5]. To neutralize the negative charges, hydrated cations (Na^+^, Ca^2+^) were electrostatically adsorbed inside the interlayer space and on the surface of the MMT. Therefore, the natural state of the MMT was a hydrophilic character, which was inherently incompatible with the vast majority of organic polymers [6]. The poor affinity between the hydrophilic MMT and the hydrophobic polymer will not result in a clay-polymer nanocomposite with improved properties compared with the pure polymer matrix. To obtain the highest enhancement of properties, the surface modification of the MMT was desirable to increase the interfacial interactions between the MMT and the polymer matrix [7]. The surface treatment not only enhanced the compatibility of the MMT with the organic polymer, but also enlarged the interlayer space between the layers of MMT. 

The most traditional and convenient method for the MMT modification was ion exchange reaction with quaternary alkylammonium salts [8]. However, the ammonium surfactants could not provide an effective thermal stability for high temperature applications because the decomposition of the ammonium surfactants could be initiated above 200 °C [9]. Thus, the modification of the MMT with phosphonium surfactants has attracted much interest from researchers. Several research papers found that the thermal stability of organoclays modified with phosphonium surfactants was superior to that of organoclays modified with ammonium surfactants [10,11]. On the other hand, the anionic surfactant was also used to improve the thermal stability of the organoclays [12]. However, it could be rather difficult to significantly expand the interlayer space of the MMT via the anionic surfactant alone. Therefore, a novel kind of organo-montmorillonite (OMMT) by the synergistic effect between the anionic and cationic surfactants has been prepared in recent years [13]. Another method for the modification of the MMT involved the use of silane coupling agents. These silanes could react with the MMT OH groups to form the chemical bonds with a covalent character between the molecules of the silane and the surface of the MMT [14]. The grafting reaction mainly occurred at the broken edges and at the structural defects situated at the external surfaces of the MMT layers [15]. However, the employment of both a phosphonium surfactant and an anionic surfactant, or both a phosphonium surfactant and a silane coupling agent, to fabricate the OMMT to increase the dispersion degree in the polymer matrix was rarely reported. 

Polystyrene (PS) is a synthetic thermoplastic that is widely used in industry due to its excellent performance of high abrasion resistance, strong plasticity and load bearing capacity [16]. However, the relatively weak thermal stability and heat resistance of PS has become a significant challenge, hindering its further applications in a variety of areas. To compensate this demerit, the addition of inorganic clay to the PS matrix can be further helpful to enhance the properties of the PS matrix [17]. Recently, an attractive technique to synthesize the clay-polymer nanocomposite is the emulsion polymerization due to the great advantage of controlled morphology and employment of water as a dispersion medium [18]. However, the surfactants were usually used in emulsion polymerization to provide colloidal stability, which may affect the final properties of the nanocomposite. The soap-free emulsion polymerization is a clean and effective way to prepare clay-polymer particles due to the use of little to no surfactant (below critical micelle concentration) in such a polymerization system [19,20]. Though the soap-free emulsion polymerization was extensively studied for the preparation of clay-polymer nanocomposites, a few papers related to the investigation of the effect of doubly organo-modified montmorillonite on the final properties of the resulting PS/OMMT nanocomposites have been published so far. 

In this work, two kinds of doubly organo-modified MMT were successfully prepared by using both a cationic surfactant and an anionic surfactant or both a cationic surfactant and a silane coupling agent. Then, the PS/OMMT nanocomposites were produced using styrene monomer and the obtained OMMT samples via the soap-free emulsion polymerization. The obtained PS/OMMT nanocomposites were dispersed into polyalphaolefin (PAO) to create lubricants. The effect arising from the doubly organo-modified MMT on the structure, morphology, thermal, and tribological properties of the PS/OMMT nanocomposites were studied and evaluated. 

## 2. Materials and Methods 

### 2.1. Materials

The natural sodium montmorillonite (MMT, cationic exchange capacity (CEC) of 100 mmol/100 g) was provided by Huai An Saibei Technology Co. Ltd., Zhangjiakou, China. The tributyltetradecylphosphonium chloride (TTPC, 97%), sodium lauryl sulfonate (SLS, 99%), and γ-methacryloxypropyltrimethoxysilane (KH570, 97%) were purchased from Aladdin Industrial Corporation, Shanghai, China. Styrene (St, 98%), divinylbenzene (DVB, 80%), methacrylic acid (MAA, 98%), and potassium persulfate (KPS, 99%) were supplied by the Tianjin Guang fu Fine Chemicals Research Institute, Tianjin, China. The St was washed with NaOH solution to remove the polymerization inhibitors, and then distilled under reduced pressure before use. Polyalphaolefin (PAO, 99% purity) was provided by Shanghai Fox Chemical Technology, Shanghai, China. Other reagents were of analytical grade and used as received. Deionized water was applied for all experiments.

### 2.2. Preparation of OMMT

#### 2.2.1. Preparation of P-MMT

**Figure 1:**
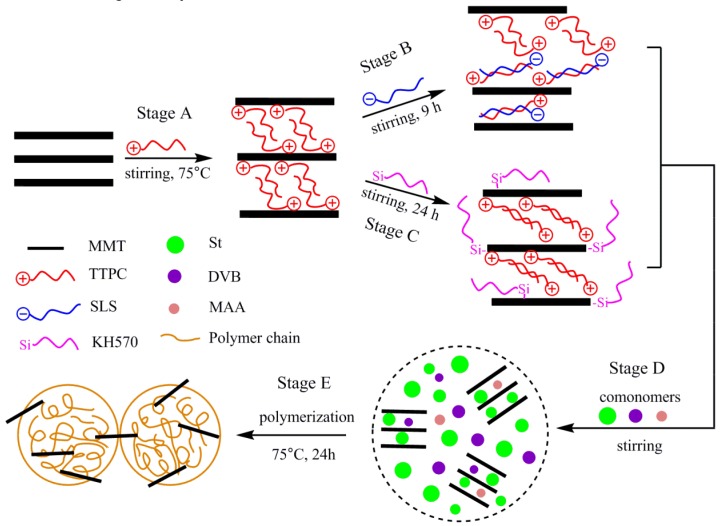
Schematic representation of the processes of preparing the organo-montmorillonite (OMMT) and the polystyrene (PS)/OMMT nanocomposites.

The modification of MMT with TTPC was carried out by the method reported in a previous paper [21]. Briefly, 2.5 g of MMT was dispersed into 75 mL of deionized water with stirring for 30 min at 75 °C. Then, 25 mL of aqueous solution containing the TTPC surfactant equivalent to 1.2 CEC of MMT was slowly added into the clay dispersion. The mixture was further stirred at 75 °C for 11 h and then filtered by vacuum filtration followed by washing with deionized water several times to remove excess TTPC molecules. The final product was dried at 60 °C for one day in a vacuum oven then ground into powder. The sample formed was referred to as P-MMT. The preparation process was shown in Figure 1 (Stage A).

#### 2.2.2. Preparation of P-SLS-MMT

The P-MMT was further modified using the SLS surfactant to produce the doubly organo-modified MMT (P-SLS-MMT) as shown in Figure 1 (Stage B) [22]. In detail, 2.5 g of P-MMT and 75 mL of deionized water were introduced in the reaction flask and stirred for 0.5 h at 75 °C. Then, the desired amount of SLS surfactant was dissolved in 25 mL of deionized water and added slowly into the suspension. The amount of SLS used was equivalent to 0.6 CEC of MMT. The mixture was stirred vigorously for 9 h at 75 °C. Finally, the resultant product was separated by centrifugation and washed four times using a mixture of ethanol/deionized water to remove the residual surfactants. The final product was dried at 60 °C overnight to obtain the P-SLS-MMT sample.

#### 2.2.3. Preparation of P-KH570-MMT

The silane functionalization of P-MMT was carried out following the method described in a previous report [23]. In a typical experiment (Figure 1 (Stage C)), the KH570 (2.483 g) was dissolved into methanol (90 mL)/deionized water (10 mL) solution with stirring, and the pH of the mixture was adjusted to 4.0 with acetic acid. The mixture was stirred for 1 h at room temperature to obtain the hydrolyzed KH570. Then, 2.5 g of P-MMT was introduced into the mixture and the suspension was further mechanically stirred with reflux at 75 °C for 24 h. Finally, the product was filtered and washed with methanol to remove the undesired siane molecules, and then dried under a vacuum oven at 60 °C for 24 h. The collected product was named as P-KH570-MMT.

### 2.3. Preparation of PS/OMMT Nanocomposite

The soap-free emulsion polymerization was applied to prepare the PS/OMMT nanocomposites according to previous reports [24,25]. Briefly, a mixture of OMMT (0.6 g, 3.0 wt % based on St), St (20.0 g), DVB (1.8 g) and an auxiliary monomer MAA (1.0 g) was ultrasonicated for 10 min in an ice bath and then magnetically stirred at room temperature for 1 h (Figure 1 (Stage D)). Meanwhile, 0.1 g of SLS was dissolved in 180 mL of deionized water with stirring for 30 min under nitrogen atmosphere. Both were later mixed in a 500-mL round-bottomed flask and mechanically stirred at 75 °C for 30 min under nitrogen atmosphere. Then, 0.109 g of KPS initiator was dissolved in 40 mL of deionized water and slowly introduced into the reactor. The resulting mixture was polymerized at 75 °C for 24 h under nitrogen atmosphere and mechanical stirring (Figure 1 (Stage E)). The final products were obtained by centrifugation and washing three times using excess hot ethanol to remove the unbound polymers, then dried overnight at 60 °C. The nanocomposite prepared with P-MMT, P-SLS-MMT, and P-KH570-MMT was abbreviated as PS/P-MMT, PS/P-SLS-MMT, and PS/P-KH570-MMT, respectively.

### 2.4. Preparation of the PAO-PS/OMMT Lubricant

The PAO-PS/OMMT lubricant was prepared to investigate the tribological performance of the PS/OMMT nanocomposites as additives. The PS/OMMT nanocomposites (1.0 wt % based on PAO) were added to the PAO, and the mixture was mechanically stirred at 1000 rpm for 30 min followed by ultrasonication for 1 h at room temperature to allow the dispersion and homogenization of the nanocomposites in the PAO. 

### 2.5. Characterization

Fourier-transform infrared (FTIR) spectra were conducted over the range of 4000–400 cm^−1^ using an FTS-3000 spectrophotometer (American Digilab) via a KBr pressed disk technique at room temperature. 

X-ray diffraction (XRD) measurements were carried out with a Bruker D8 Advance X-ray Diffractometer (Stadi P, Karlsruhe, Germany) using Cu-Ka radiation (λ = 0.1540 nm) with a scanning rate of 2°/min, 40 kV, and 30 mA over the 2θ range of 1.5°–20° by a scanning interval of 0.02°. The corresponding basal space was computed by applying the Bragg's Law. 

Dynamic light scattering (DLS) was performed using a Malvern Mastersizer 2000 (Malvern Instruments, Malvern, UK) to determine the particle size and size distribution of the samples at room temperature. 

Scanning electron microscopy (SEM, SU8010, Hitachi, Tokyo, Japan) coupled with an energy dispersing spectrometer (EDS) was used to study the surface morphology and elements of the nanocomposites. The samples were coated with a thin layer of gold prior to SEM observation. The EDS analysis was registered three times in different points, and the result was the average of three obtained values. 

Transmission electron microscopy (TEM) analyses were performed on a JEM-2100 TEM operating at an accelerating voltage of 200 kV. 

Thermogravimetric analysis (TGA) was carried out on a NETZSCH thermogravimetric analyzer (TGA, STA409PC, Bavaria, Germany) at a heating rate of 10 °C/min from 25 °C to 800 °C under a nitrogen flow. Differential thermogravimetry (DTG) curves were calculated from the TGA results. 

The tribological measurements were performed using a four-ball tester (MRS-1J, Jinan, China) under a load of 392 N at a rotating speed of 1450 r/min for 1 h at room temperature. Prior to testing, the steel bearing balls GCr15 (diameter of 12.7 mm, grade 10) were cleaned ultrasonically for 30 min with ethanol. The average values of coefficient of friction (COF) were reported from three measurements of each sample.

## 3. Results

### 3.1. Surface Treatments of Montmorillonite

#### 3.1.1. FTIR Analysis

**Figure 2:**
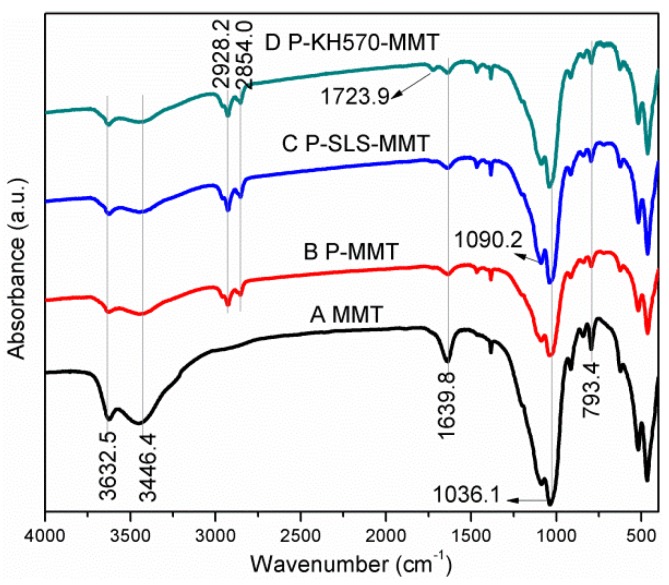
Fourier-transform infrared (FTIR) spectra of (**A**) sodium montmorillonite (MMT), (**B**) P-MMT, (**C**) P-SLS-MMT, and (**D**) P-KH570-MMT.

The FTIR spectra of MMT, PMMT, P-SLS-MMT, and P-KH570-MMT are given in Figure 2. In the spectra of MMT, the broad absorption bands at 3446.4 and 1643.4 cm^−1^ corresponded to the –OH stretching vibration and H–O–H bending vibration of adsorbed water within MMT interlayer, respectively. The characteristic absorption peak at 3632.5 cm^−1^ was attributed to the stretching vibration of the Al–OH and Si–OH in the silicate layers, and this –OH group was responsible for the reaction with the silane coupling agent [26]. The bands at 1036.1 and 793.4 cm^−1^ were representative of the stretching vibration of Si–O and Si–O–Al, respectively. In addition of these characteristic peaks of MMT, novel vibration bands related to surfactants and/or silane coupling agents appeared in the spectra of OMMT (Figure 2B–D). For the P-MMT, the asymmetric and symmetric stretching vibration of –CH_2_ of phosphonium surfactants was observed at 2928.2 and 2854.0 cm^−1^, respectively. This is direct evidence for the successful intercalation of TTPC surfactants into the interlayer space of MMT. In comparison with the P-MMT, the intensity of the absorption bands at 2928.2 and 2854.0 cm^−1^ increased for the P-SLS-MMT, which can be reasonably attributed to the introduction of the SLS surfactants. Besides, the absorption band at 1090.2 cm^−1^ corresponded to the asymmetric stretching vibration of –SO_3_H group of SLS surfactants. These results indicated that the TTPC and SLS surfactants were synergistically incorporated into the interlayer space of MMT. The spectrum of P-KH570-MMT not only had the characteristic bands of P-MMT, but also exhibited a novel band at 1723.9 cm^−1^, which was assigned to the stretching vibration of the C=O in the KH570 molecules. In addition, higher intensity of the absorption bands at 2928.2 and 2854.0 cm^−1^ were also found for the P-KH570-MMT sample, implying that KH570 was successfully grafted on the surfaces and edges of the silicate layers [27]. These observations suggested that the MMT was doubly organo-modified by a phosphonium surfactant and a silane coupling agent. It is worth mentioning that a significant decrease of the relative intensity of the bands at 3632.5–3446.4 cm^−1^ and 1639.8 cm^−1^ was observed for the OMMT compared with the MMT, which indicated that the surface of OMMT changed from a hydrophilic to a hydrophobic character and the content of water over OMMT decreased. The above results indicated that the target doubly organo-modified MMT was successfully synthesized. 

#### 3.1.2. XRD Analysis

**Figure 3:**
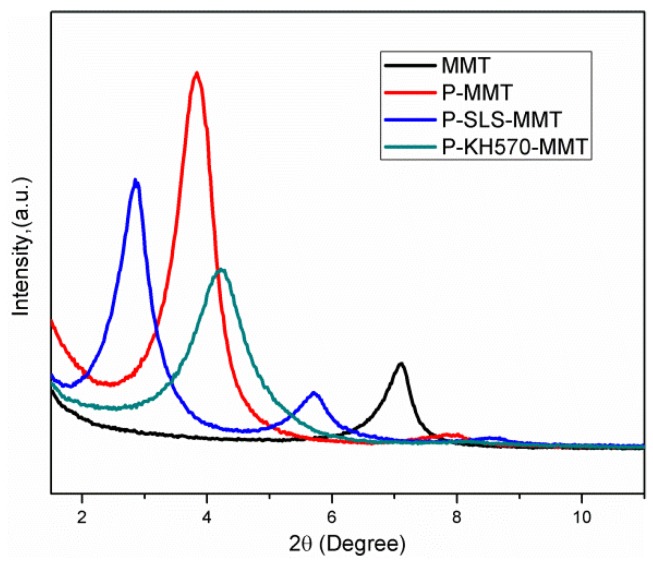
XRD patterns of MMT, P-MMT, P-SLS-MMT, and P-KH570-MMT.

The XRD patterns obtained for the MMT and OMMT are presented in Figure 3. The MMT showed a typical XRD reflection at 2θ = 7.28°, corresponding to a basal spacing of 1.21 nm according to Bragg's law. This value is in agreement with similar hydrated clays reported in the literature [28]. The significant increase in the interlayer space of the MMT was observed after the modification with surfactants. In the case of P-MMT, the characteristic peak shifted from 7.28° to 3.84°, corresponding to an increase in basal space from 1.21 nm to 2.36 nm. This result confirms that the intercalation of phosphonium surfactant was really achieved, which is consistent with the FTIR results. In the XRD spectrum of P-SLS-MMT, the distribution of interlayer space of platelets was characterized by two prominent reflections that appeared at 2θ = 2.87° and 5.79°, corresponding to interlayer spaces of 3.07 nm and 1.52 nm, respectively. The basal space of P-SLS-MMT was 0.71 nm higher than that of P-MMT, which indicated that SLS successfully entered the interlayer space of P-MMT and cation-anion type OMMT was prepared. The anionic surfactants can form ion-pairs with the cationic surfactants to intercalate into the interlayer space of MMT. This result was also observed by other researchers [29,30]. The appearance of a second peak in the XRD pattern of P-SLS-MMT was attributed to the (002) reflection. The diffraction peak of P-KH570-MMT at 4.21° corresponded to an interlayer space of 2.09 nm. Compared with P-MMT, the decrease in the interlayer space of P-KH570-MMT was mainly due to partly intercalated cationic surfactants becoming washed out with the use of hydroalcoholic solution as a solvent in the silylation reaction. This result indicated that the KH570 was mainly grafted onto the broken edges and external surfaces or undergoing ion exchange with the pre-existing surfactants in a very low amount, thus merely had a significant impact on the interlayer structure of P-MMT [31].

#### 3.1.3. TGA and DTG Analysis

**Figure 4:**
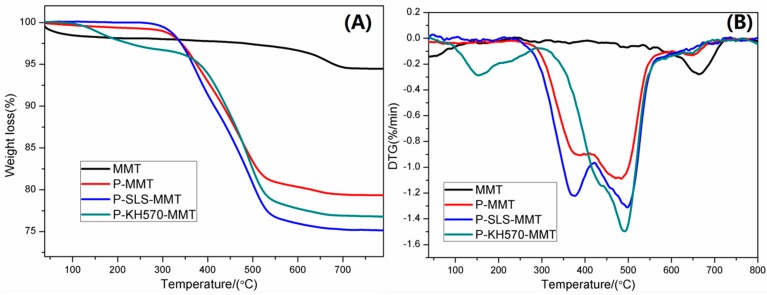
(**A**) Thermogravimetric analysis (TGA) and (**B**) Derivative thermogravimetry (DTG) curves of MMT, P-MMT, P-SLS-MMT, and P-KH570-MMT.

Figure 4 shows the thermogravimetric analysis (TGA) and derivative thermogravimetry (DTG) curves of MMT, PMMT, P-SLS-MMT, and P-KH570-MMT samples. In Table 1, the mass loss associated with the different temperature ranges is listed. Two distinct thermal degradation events were obtained in the DTG curves of the MMT. The first mass loss, occurring at a temperature of 75 °C, was assigned to the elimination of physically adsorbed water, and the second at 660 °C corresponded to the dihydroxylation of clay layers [32]. The mass loss of OMMT was higher than that of MMT at 200–600 °C, which was evidence of intercalated surfactants and/or grafted silane coupling agents. For the P-MMT, the mass loss below 200 °C was lower when compared with the MMT, implying less adsorbed water in the P-MMT. Furthermore, the mass loss of the P-MMT between 200 °C and 600 °C was obviously higher than that of the MMT, which was attributed to the degradation of adsorbed surfactant molecules on the external surface and of intercalated surfactant molecules in the interlayer space of the MMT. The overall mass loss of P-MMT (20.7 wt %) was higher than that of MMT (5.6 wt %). Compared with P-MMT, a greater mass loss in the range of 200 °C to 600 °C was found in P-SLS-MMT, which could be attributed to the intercalation of SLS molecules into the interlayer space of P-MMT. In addition, the maximum mass loss rate temperature of P-SLS-MMT occurred at 499 °C, higher than 15 °C in P-MMT, indicating the thermal properties of P-MMT were enhanced owing to the introduction of the SLS molecules. These results suggested that the SLS molecules were successfully intercalated into the interlayer space of the P-MMT. This result was consistent with the results of XRD analysis. In the case of P-KH570-MMT, the first mass loss occurred at a temperature below 200 °C which was attributed to the evaporation of adsorbed water and/or of free molecules of solvent in the pores of MMT. The mass loss observed over the range of 200–600 °C was assigned to the loss of surfactant molecules combined with the decomposition of adsorbed and/or grafted KH570 molecules introduced during the silylation reaction. Compared with the P-MMT, the observed increase in mass loss for the P-KH570-MMT between 200 °C and 600 °C was attributed to the intercalated and/or grafted silane molecules. Combining this with the result of XRD, the interlayer space of P-KH570-MMT was smaller than that of P-MMT, the increasing amount of KH570 molecule was mainly grafted on the broken edges and the external surface of MMT, not intercalating in the interlayer space of MMT [33]. 

### 3.2. Characterization of PS/OMMT Nanocomposite 

#### 3.2.1. FTIR Analysis

**Figure 5:**
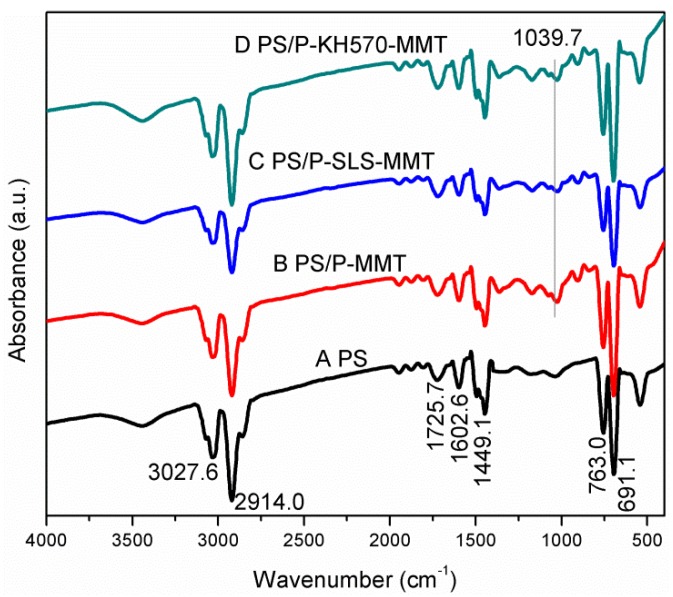
FTIR spectra of (**A**) PS, (**B**) PS/P-MMT, (**C**) PS/P-SLS-MMT, and (**D**) PS/P-KH570-MMT.

The FTIR spectra of the PS, PS/P-MMT, PS/P-SLS-MMT, and PS/P-KH570-MMT are shown in Figure 5. In the case of PS, the characteristic bands at 3027.6 and 2914.0 cm^−1^ were attributed to the CH aromatic stretching vibrations and CH_2_ asymmetric stretching vibrations, respectively. Two absorption bands at 1602.6 and 1449.1 cm^−1^ were ascribed to the stretching vibration of the aromatic C=C. There were two strong absorption bands at 763.0 and 691.1 cm^−1^ that corresponded to the CH out-of-plane deformation of benzene rings. In addition, the peak at 1725.7 cm^−1^ was due to the C=O stretching vibration of methacrylic acid. These observations indicate that the polymerization reaction of styrene monomer has been occurred [34]. The FTIR spectra of PS/P-MMT not only had the characteristic bands of PS, but also exhibited new absorption bands at 1039.7 cm^−1^, which were assigned to Si-O stretching vibrations of the MMT. This result proved the formation of PS/P-MMT nanocomposites. The presence of the characteristic peak of MMT in the polymer matrix was also observed in the samples of PS/P-SLS-MMT and PS/P-KH570-MMT.

#### 3.2.2. XRD Analysis

**Figure 6:**
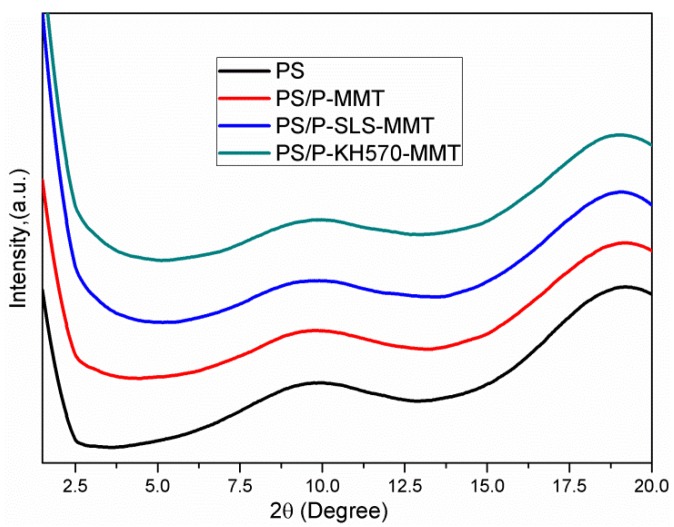
XRD patterns of PS, PS/P-MMT, PS/P-SLS-MMT, and PS/P-KH570-MMT.

**Figure 7:**
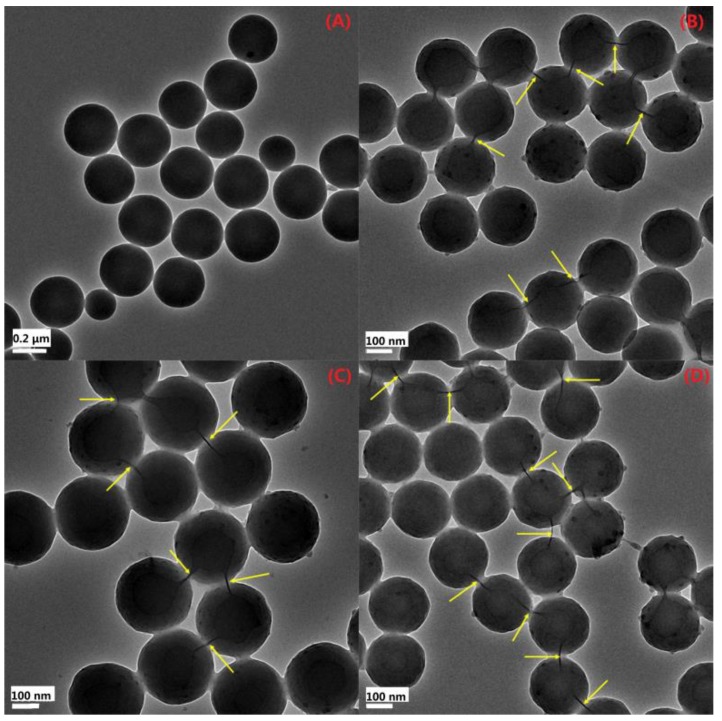
Transmission electron microscopy (TEM) images of (**A**) PS, (**B**) PS/P-MMT, (**C**) PS/P-SLS-MMT, and (**D**) PS/P-KH570-MMT.

Figure 6 shows the XRD patterns of the PS, PS/P-MMT, PS/P-SLS-MMT, and PS/P-KH570-MMT nanocomposites. As shown in Figure 6, no characteristic diffraction peak of OMMT was observed for the PS/P-MMT, PS/P-SLS-MMT, or PS/P-KH570-MMT in the range of 1.5°–10.0° of 2θ. This result suggested that the PS chains entered into the interlayer space of OMMT and destroyed the intercalated clay structure, leading to exfoliated structure in the resulting PS/OMMT nanocomposite during the soap-free emulsion polymerization. The clay platelets could be exfoliated in the PS matrix owing to the role of the intercalated phosphonium surfactants, SLS molecules, and/or adsorbed KH570 silanes that obviously enlarged the interlayer space of the MMT, which could provide a bigger space for the ease of PS macromolecular chains penetration. In addition, the complete exfoliation of individual clay platelets in the polymer matrix was also because of the favorable miscibility between the organic clay platelets and polymer matrix [35]. Furthermore, the KH570 molecules that grafted on the broken edges of the MMT could improve the hydrophobicity of the layered silicates, thus increasing their compatibility with the PS matrix [36]. However, the disappearance of XRD peaks does not necessarily suggest complete delamination of the clay platelets. Thus, other analytical tools, such as TEM, were needed to prove the dispersion and degree of exfoliation of the clay platelets. In order to determine the deductions from XRD results, the morphology of the nanocomposites was further analyzed by the use of TEM. Figure 7 shows the typical TEM images of the PS and PS/OMMT nanocomposites. 

#### 3.2.3. TEM Analysis

As shown in Figure 7A, spherical particles could be observed for the pure PS. However, the size distribution of particles was broad, and the particle size was in the range of approximately 200–600 nm. In contrast, when the OMMT was introduced into the polymer matrix, the particle sizes of the PS/OMMT nanocomposites became much smaller, as shown in Figure 7B–D. Moreover, the size distribution of PS/OMMT particles was narrow and monomodal, and the particle size was approximately between 250 nm and 400 nm. These results indicated that the morphology of the polymer particles was changed by the presence of OMMT. The reason was probably that the movement of the styrene monomers and growth of the PS chains were effectively reduced via the shielding effects of the clay platelets. As denoted by the yellow arrows in Figure 7B–D, the existence of exfoliated clay layers encapsulated inside the polymer particles was clearly visible. The TEM images of PS/OMMT nanocomposites showed that the nanoclay layers were completely exfoliated and dispersed homogeneously within the PS matrix. It showed that a highly exfoliated structure of PS/OMMT nanocomposite was obtained, which was in accordance with the corresponding XRD results. This result implied that the clay layers of 3 wt % OMMT could maintain the polymer colloidal stability, thus resulting resulted in the complete exfoliation of the clay layers and formation of exfoliated structure of PS/OMMT nanocomposites [37]. 

#### 3.2.4. DLS Analysis

**Figure 8:**
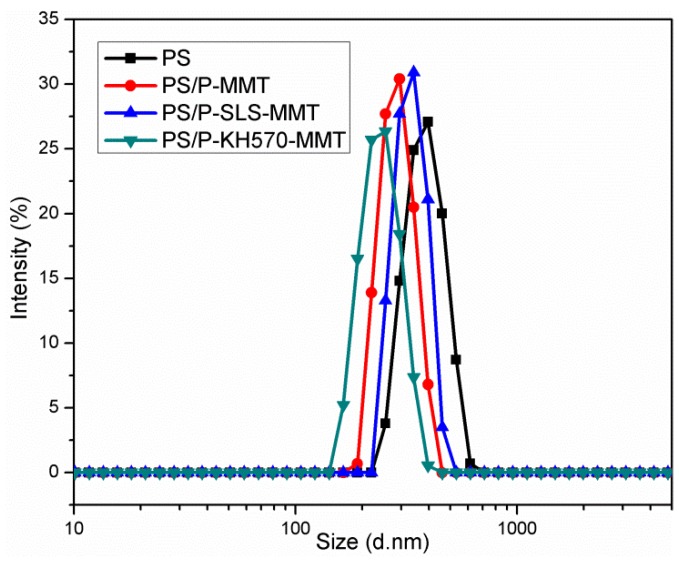
Size distribution curves of PS, PS/P-MMT, PS/P-SLS-MMT, and PS/P-KH570-MMT.

Figure 8 shows the size distributions of the pure PS and PS/OMMT nanocomposite samples, and the average particle size data and PDI values are summarized in Table 2. The particle size distributions of all samples showed only one narrow peak, and the average diameters of the polymer particles were in the range of 241.3–378.6 nm. These results were consistent with the results of the TEM analysis. The average particle size of the pure PS was 378.6 nm, while the average particle sizes of the PS/OMMT nanocomposites were 290.4 nm, 338.8 nm, and 241.3 nm for the PS/P-MMT, PS-P-SLS-MMT, and PS/P-KH570-MMT, respectively. The decrease in the average particle size of the nanocomposites was owing to the addition of OMMT would more likely to facilitate the nucleation to form smaller and more uniform particles. On the other hand, the PDI values, which were indicative of the monodispersity of polymer particles, were observed to decrease for the PS/OMMT nanocomposites compared with the pure PS. This observed decrease was owing to the self-arrangement of the modified MMT layers within the PS particles might affect the surface tension and cause fine and stable particles. Moreover, the hydrophobic MMT layers could act as stabilizers to improve the monodispersity of the PS particles [38]. The organic modification of MMT improved its compatibility with the monomer phase and promoted the exfoliation of the MMT, which was beneficial to the encapsulation of clay platelets by the polymer particles. The smallest particle size and PDI value were obtained for the PS/P-KH570-MMT sample, which was due to the ability of the vinyl group of the KH570 molecule to participate in the polymerization reaction. 

#### 3.2.5. SEM-EDS Analysis

**Figure 9:**
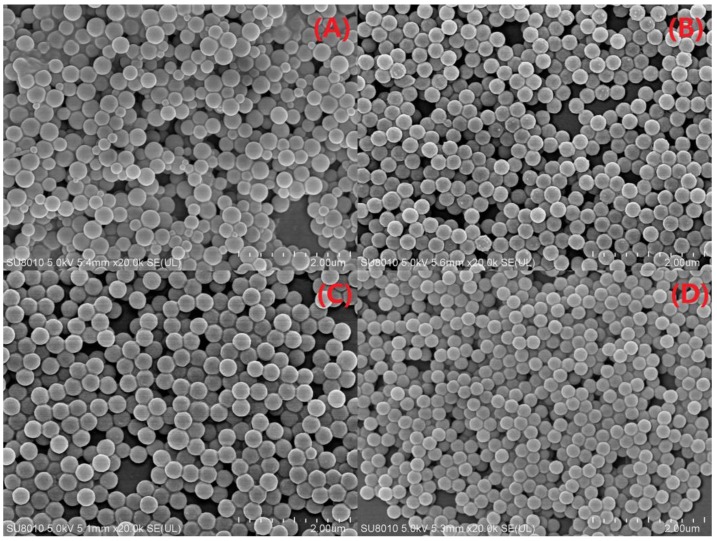
SEM images of (**A**) PS, (**B**) PS/P-MMT, (**C**) PS/P-SLS-MMT, and (**D**) PS/P-KH570-MMT.

Figure 9 shows the SEM images of the PS, PS/P-MMT, PS/P-SLS-MMT, and PS/P-KH570-MMT nanocomposites. As seen in Figure 9A, the particles of pure PS were mostly spherical and the particle size was approximately 380 nm with a broad size distribution. In particular, a certain degree of coagulation of the particles was observed due to the poor stability. Compared with the pure PS, the particle morphology of the PS/OMMT nanocomposites was changed by the presence of the OMMT. When the OMMT was introduced in Figure 9B–D, the particle size distribution became narrow, and the particle size decreased to 250–350 nm. These results were in good agreement with the results of TEM and DLS. Moreover, each PS/OMMT particle kept good dispersion stability because of the stabilizing effect of OMMT for the polymer particle. 

The results of the SEM-EDS analysis are given in Table 3. The most pronounced distinction between the pure PS and the PS/OMMT nanocomposites was the drastic increases in the amount of Al and Si content in the PS/OMMT samples. This result indicated that the OMMT was successfully introduced in the polymer matrix. 

#### 3.2.6. TGA and DTG Analysis

**Figure 10:**
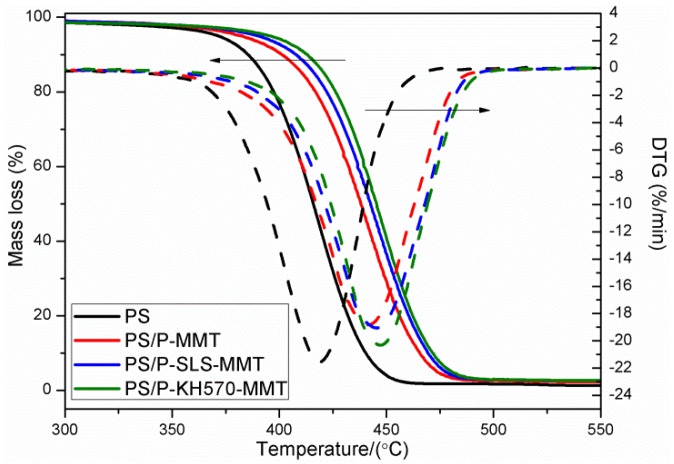
TGA and DTG curves of PS, PS/P-MMT, PS/P-SLS-MMT, and PS/P-KH570-MMT.

Figure 10 presents TGA and DTG curves for the pure PS, PS/P-MMT, PS/P-SLS-MMT, and PS/P-KH570-MMT. It can be seen from the TGA curves that the weights of residue left of PS/OMMT nanocomposites at 550 °C were increased compared with the pure PS, which indicated the presence of the MMT in the polymer matrix. Furthermore, in contrast to the pure PS, the PS/OMMT nanocomposites exhibited significantly improved thermal stability, which could be attributed to the addition of OMMT in the polymer matrix [39]. The onset decomposition temperature corresponding to a mass loss of 10 wt % (*T*_10%_) of pure PS was 383 °C. As for the PS matrix filled with OMMT, the onset decomposition temperatures of PS/P-MMT, PS/P-SLS-MMT, and PS/P-KH570-MMT were about 399 °C, 406 °C, and 416 °C, respectively, that was, 16 °C, 23 °C, and 33 °C higher than that of pure PS. The maximum mass loss temperature (*T*_max_, observed from DTG) was raised from 419 °C for the pure PS to 440 °C, 443 °C, and 449 °C for PS/P-MMT, PS/P-SLS-MMT, and PS/P-KH570-MMT, respectively. The enhancement in the thermal stability was due to the MMT layers forming a three-dimension network and a labyrinth pathway in the clay-polymer nanocomposite to prevent out-diffusion of the volatile decomposition products during thermal decomposition [40]. Meanwhile, the exfoliated structure of the MMT worked as a mass transport barrier, or a nucleating agent in the matrix, by limiting the heat transfer and absorbing the heat to decelerate the degradation of the polymers [41]. However, it was observed that the *T*_10%_ and *T*_max_ of PS/P-SLS-MMT were higher than those of PS/P-MMT. The reason was perhaps that the maximum mass loss temperature of P-SLS-MMT was higher than that of P-MMT, as shown in Figure 4B, due to the electrostatic interaction between the anionic and cationic surfactants. This improvement in thermal stability was also observed in the PS/P-KH570-MMT nanocomposite. These results revealed that the double-modified MMT-containing PS nanocomposites showed higher thermal stability than that of the single-modified one. Interestingly, the *T*_10%_ and *T*_max_ of the PS/P-KH570-MMT were higher than those of the PS/P-SLS-MMT. This result indicated that the double bonds of silanes grafting on the edges and surfaces of the MMT could react with styrene monomer increased chemical interaction between the MMT and the polymers, resulting in the improved interface interactions and enhanced the thermal stability [42]. In this case, the PS chains were covalently bonded on the surface of MMT, and the grafted KH570 molecules worked as the “bridge” connecting the PS chains and silicate layers, leading to an increase in the thermal stability. 

#### 3.2.7. Friction Performance Analysis

**Figure 11:**
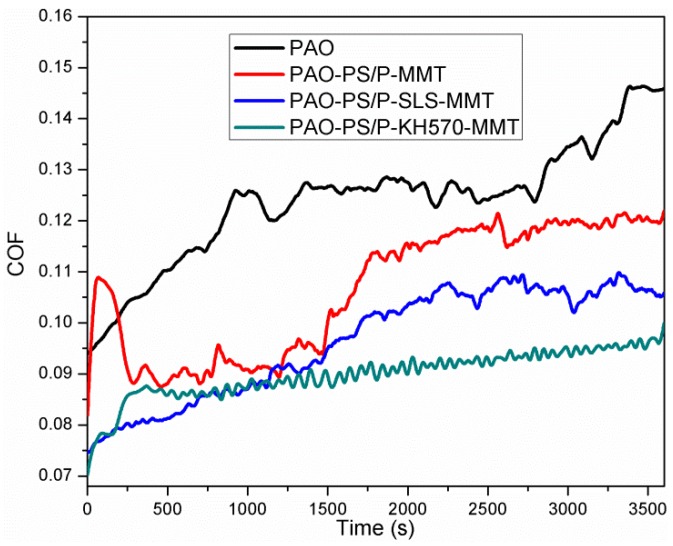
The COF versus time of PAO, PAO-PS/P-MMT, PAO-PS/P-SLS-MMT, and PAO-PS/P-KH570-MMT.

Figure 11 shows the coefficient of friction (COF) versus time of PAO and PAO with PS/OMMT nanocomposite samples. As it can be seen from the figure, the COF of PAO was around 0.095 at the beginning and then increased dramatically to about 0.125 within 1000 s, followed by a stable period of approximately 2700 s. The COF then increased from 0.120 to about 0.145 in the rest of the test. For PAO-PS/P-MMT, the COF rose up sharply from 0.082 to 0.110, dropped drastically to 0.090 within 250 s, and then experienced a stable period with fluctuation around 1500 s. The COF then increased slowly to 0.115 at the end of the test. The COF of PAO-PS/P-SLS-MMT started at 0.070, increased slowly to about 0.100 around 2200 s, and then remained steady during the rest of the test. The COF of PAO-PS/P-KH570-MMT was 0.070 at the beginning, increased to 0.085 within 250 s, and then rose up slowly. At the end of the test, the COF reached 0.090. It can be found from the test results that the adding PS/OMMT nanocomposites to the PAO decreased the COF values and clearly enhanced the friction reduction properties. Furthermore, with the same content of nanocomposite particles, PS/P-KH570-MMT performed the lowest COF value and the best friction reduction properties.

**Figure 12:**
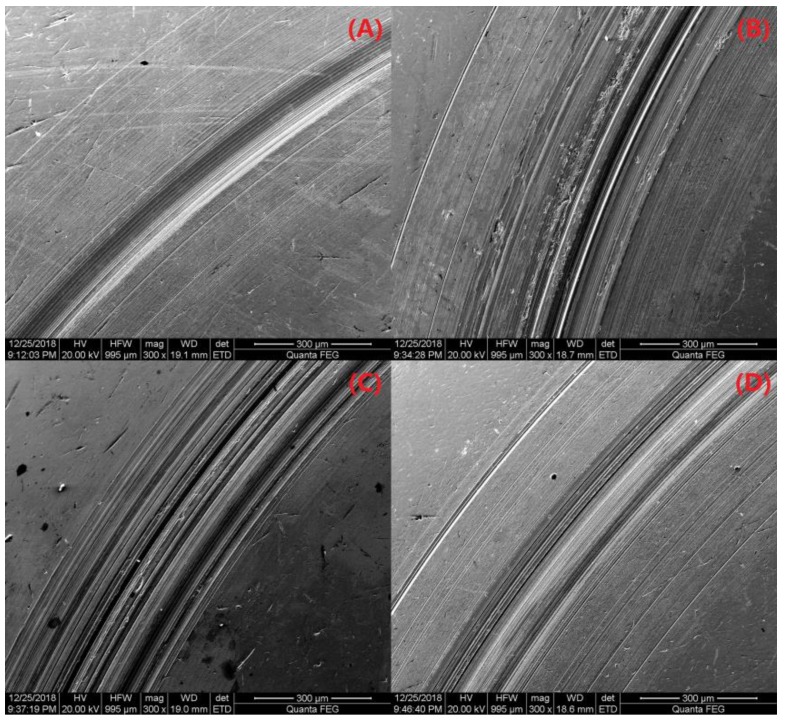
SEM images of the upper ball of (**A**) PAO, (**B**) PAO-PS/P-MMT, (**C**) PAO-PS/P-SLS-MMT, and (**D**) PAO-PS/P-KH570-MMT.

**Figure 13:**
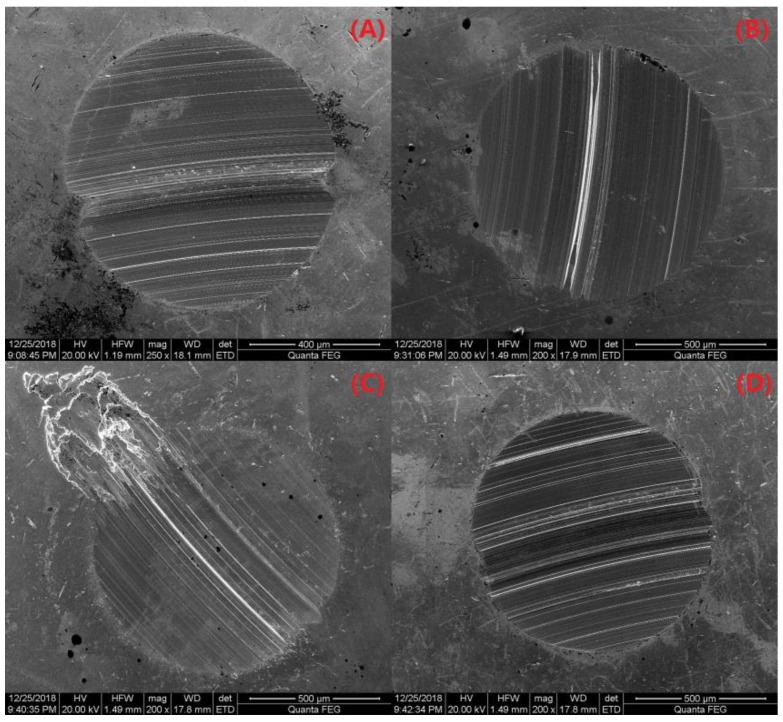
SEM images of the lower ball of (**A**) PAO, (**B**) PAO-PS/P-MMT, (**C**) PAO-PS/P-SLS-MMT, and (**D**) PAO-PS/P-KH570-MMT.

To further characterize the tribological properties for the PS/OMMT nanocomposites, we examined the wear surfaces of the PAO and PAO-PS/OMMT nanocomposites using SEM. Figure 12A–D and Figure 13A–D show the SEM morphology of the wear surfaces of the upper and lower ball of the four-ball machine for PAO, PAO-PS/P-MMT, PAO-PS/P-SLS-MMT, and PAO-PS/P-KH570-MMT, respectively. From Figure 12A, a wide and deep plowing groove was found on the wear surface, indicating serious abrasive wear. This worn morphology was also observed in Figure 13A. However, when the PS/OMMT nanocomposites were added into the PAO, as shown in Figure 12B–D and Figure 13B–D, the grooves on the wear surface became shallower and narrower, which indicated that the surface roughness was improved and the COF was reduced. These results indicated that the PS/OMMT nanocomposites exhibited excellent anti-wear and friction reduction properties in their tribological properties in a lubricant, which were consistent with the results of Figure 11. During the lubrication process, the nanocomposite particles were able to fill in the microgrooves on the wear surfaces and reduce the roughness of the metal surfaces to reduce friction and wear [43]. Furthermore, the nanocomposite particles could support the metal surfaces and prevent them from contacting each other and roll like a bearing ball, thereby reducing the COF and wear [44]. Among the wear surfaces, there was a smoother wear surface with microgrooves and nanogrooves in Figure 12D and Figure 13D. This result suggested that the best anti-wear and friction reduction properties among all the type of nanocomposites were observed with the PS/P-KH570-MMT nanocomposite in this study. This may be due to the smaller PS/P-KH570-MMT nanocomposite particle filling the grooves more effectively than the larger particles. The nanocomposite particles with smaller diameters could interact with the metal surface more effectively and form the transfer film more easily to protect the metal surface and reduce the wear. Meanwhile, with higher thermal stability, the PS/P-KH570-MMT nanocomposite particles might roll better between two wear surfaces and reduce the friction. The above results indicated that the improvements of tribological properties of PS/OMMT nanocomposite particles showed promising applications in the drilling fluid lubrication. 

## 4. Conclusions

Novel PS/OMMT nanocomposite particles were synthesized by soap-free emulsion polymerization in the presence of doubly organo-modified MMTs and investigated. The anionic surfactant, SLS, could be effectively used to further increase the interlayer space of P-MMT by the electrostatic interaction. The silane coupling agent, KH570, could be introduced to the external surfaces and broken edges of the P-MMT due to the silylation reaction. The complete exfoliation of the OMMT within the PS matrix was confirmed by XRD and TEM analysis. The introduction of OMMT significantly decreased the average particle size and PDI values of PS, as evidenced by DLS and SEM results, and this became more pronounced in the PS/OMMT nanocomposites filled with P-KH570-MMT. The results of TGA-DTG analysis confirmed that the thermal stability of the PS/OMMT nanocomposites was significantly enhanced compared with pure PS. The *T*_max_ increased from 419 °C of pure PS to 449 °C of PS/P-KH570-MMT. Moreover, the COF values of PAO-PS/OMMT nanocomposites were well below than that of pristine PAO, indicating increased friction reduction properties. Available results in this work can provide significant guidance for the future synthesis and application of the PS/OMMT nanocomposites in the oil and gas drilling engineering field to improve drilling fluid lubrication.

## Figures and Tables

**Table 1:** Relative mass loss during decomposition of investigated samples (wt %).

Sample	Temperature Range		
	<200 ± 1.5 °C	200–600 ± 1.9 °C	30–800 ± 2.2 °C
MMT	1.9	1.5	5.6
P-MMT	0.6	19.0	20.7
P-SLS-MMT	0.2	23.9	24.9
P-KH570-MMT	2.3	19.9	23.3

**Table 2:** Particle size distribution of PS, PS/P-MMT, PS/P-SLS-MMT, and PS/P-KH570-MMT samples.

Sample	Z-Average (nm)	PDI
PS	378.6 ± 8.9	0.232
PS/P-MMT	290.4 ± 3.8	0.159
PS/P-SLS-MMT	338.8 ± 3.6	0.160
PS/P-KH570-MMT	241.3 ± 2.1	0.074

**Table 3:** Distribution of the elements in PS, PS/P-MMT, PS/P-SLS-MMT, and PS/P-KH570-MMT samples found by EDS measurement related with SEM images given in Figure 9.

Sample	C (wt %)	O (wt %)	Al (wt %)	Si (wt %)
PS	68.44 ± 0.46	30.33 ± 0.36	0.70 ± 0.09	0.53 ± 0.07
PS/P-MMT	63.62 ± 0.24	31.45 ± 0.40	3.52 ± 0.17	1.41 ± 0.12
PS/P-SLS-MMT	64.15 ± 0.30	31.36 ± 0.38	3.05 ± 0.19	1.44 ± 0.13
PS/P-KH570-MMT	67.92 ± 0.33	28.61 ± 0.29	2.33 ± 0.11	1.14 ± 0.12
